# Non-Steady State NMR Effect and Application on Time-Varying Magnetic Field Measurement

**DOI:** 10.3390/s22249960

**Published:** 2022-12-17

**Authors:** Xiaohu Zeng, Hong Ma, Jiang Jin, Hua Zhang, Jingwen Ma

**Affiliations:** 1 School of Electronic Information and Communications, Huazhong University of Science and Technology, Wuhan 430074, China; 2 College of Life Science and Technology, Huazhong University of Science and Technology, Wuhan 430074, China

**Keywords:** time-varying magnetic field measurement, non-steady state nuclear magnetic resonance (NSS-NMR), orthogonal dual-coil probe, directional coupler, Larmor precession relationship

## Abstract

The measurement of a time-varying magnetic field is different from a constant magnetic field, due to its field intensity variation with time. Usually, the time-varying magnetic field measurement converts the solution of the magnetic induction intensity into the calculation of the induced electromotive force (EMF); then, the magnetic induction intensity is obtained by the time integration of the EMF, but the process is vulnerable to external interference. In this paper, a non-steady state nuclear magnetic resonance (NSS-NMR) scheme for the measurement of a time-varying magnetic field is proposed. In a time-varying magnetic field environment, an RF excitation signal with a certain frequency bandwidth is applied to excite the nuclear spin system. The NSS-NMR signal, which varies with time in the frequency range corresponding to the frequency bandwidth of the RF excitation, could finally be obtained after a series of processing of the probe output signal. During the NSS-NMR experiment, an orthogonal dual-coil probe is adopted to synchronously generate the RF excitation and induce the probe output signal. Moreover, a directional coupler that utilized in the experiment outputs a reference signal from the coupling port for the subsequent signal processing. The experimental results show that the weak NSS-NMR signal is indeed observed. The longitudinal time-varying magnetic field ranges from 0.576 T to 0.582 T, which is inverted by the Larmor precession relationship, have been successfully detected based on the so-called NSS-NMR effect.

## 1. Introduction

Magnetic field measurement provides reliable field-strength information for scientific research in a magnetic field environment. The common magnetic resonance methods, including the electron paramagnetic resonance (EPR) method, optical pump magnetic resonance (OPMR) method and nuclear magnetic resonance (NMR) method [1,2,3], could be utilized to measure a constant magnetic field [4,5]. The EPR method applies the microwave radiation resonant absorption effect of the paramagnetic system but it is susceptible to external interference, so the measurement accuracy mainly depends on the experimental environment [6,7]. The OPMR method is characterized by poor stability and is sensitive to ambient temperature fluctuation and mechanical vibration. In addition, the operation of the OPMR experimental system is rather complicated, resulting in a lower measurement accuracy [8]. The NMR method utilizes the inductive absorption effect for the incident electromagnetic wave in a nuclear spin system, for which the Larmor precession frequency is proportional to the magnetic induction intensity of the longitudinal magnetic field. Theoretically, since the accuracy of the gyromagnetic ratios of the common nucleus (usually ^1^H) and frequency measurement is extremely high, the accuracy of the magnetic field measurement by the NMR method will also be very high; for example, the relative accuracy is better than 10 ppm [9]. Therefore, the NMR measurement result is often used as the magnetic field measurement benchmark to calibrate other types of magnetometers [10,11].

Conventionally, the NMR method is applied to measure the magnetic induction intensity of a permanent magnet [12]. The radio frequency (RF) pulse sequence is utilized to excite the nuclear spin system in a conventional NMR experiment, the pulse width is inversely proportional to the measurement range of the time-varying magnetic field. It suggests that the wider the magnetic field measurement range is, the shorter the pulse width of the exciting pulse sequence will be. However, the shorter pulse width means the RF pulse power should be greater than that of the wider RF pulse for the effective excitation of the nuclear spin system. Wenjun Chen et al. had suggested that a continuous band-pass RF signal could be used to excite the nuclear spin system in an NMR experiment and simulated the so-called non-steady state nuclear magnetic resonance (NSS-NMR) effect in a pulsed high magnetic field [13,14,15], but they have not verified it in practice. In this paper, the NSS-NMR based time-varying magnetic field measurement scheme is proposed and verified in practice. After the data processing of the output signal of the orthogonal dual-coil probe, the NSS-NMR signal is successfully observed, and the longitudinal time-varying magnetic field is finally inverted by the Larmor precession relationship.

In an NSS-NMR experiment, the RF excitation signal is time continuous with a certain frequency bandwidth, so a pair of wideband dual-coil probes is designed and fabricated to excite the nuclear spin system and simultaneously induce the NSS-NMR signal. In this paper, the equivalent circuit of the probe and its extraction parameters, and two kinds of dual-port remote impedance matching methods are also described in detail. The experimental results show that the NSS-NMR effect indeed exists and could be used to measure the longitudinal time-varying magnetic field.

## 2. The NSS-NMR Effect and Its Numerical Simulation Result

### 2.1. The NSS-NMR Effect

A conventional NMR experiment is performed in a constant magnetic field while the nuclear spin system is simultaneously excited by an orthogonal-polarization RF magnetic field. The exciting condition of the NMR effect is that the angular frequency of the RF magnetic excitation field $\vec{\omega }$ is equal to the Larmor precession frequency ${\vec{\omega }}_{0}$ [16], as depicted by the following Equation (1):

(1) $$\vec{\omega }={\vec{\omega }}_{0}=-\gamma {\vec{B}}_{0}$$

where ${\vec{B}}_{0}$ and γ are the longitudinal constant magnetic induction intensity and gyromagnetic ratio of the nucleus spin system, respectively. For example, the gyromagnetic ratio of ^1^H nucleus is $2.6752\times 10^{8}\;rad\cdot s^{-1}\cdot T^{-1}$.

The Bloch equations systematically describe the NMR effect in the constant magnetic field from the perspective of classical mechanics [17]. Accordingly, if the magnetic induction intensity of the longitudinal magnetic field varies from $B_{01}$ to $B_{02}$ and the angular frequency ω of the exciting RF signal synchronously covers $\omega_{1}$ (=$\gamma B_{01}$) to $\omega_{2}$ (=$\gamma B_{02}$),the NMR effect may occur throughout the duration of the RF excitation signal. Since the nuclei are in the excitation and relaxation state alternately, the Bloch equations should be appropriately modified to describe the variation of the macroscopic magnetization and the proposed NSS-NMR effect. The NSS-NMR effect means the unstable and non-steady precession and nutation in the nuclear spin system. Therefore, the meaning of the NSS-NMR effect includes two aspects; since the macroscopic magnetization varies along with the longitudinal time-varying magnetic field and the orthogonal-polarization RF magnetic field, there is no stable equilibrium state or termination state for the precession and nutation.

### 2.2. Numerical Simulation Result of the NSS-NMR Effect

In order to acquire the variety of macroscopic magnetization in the NSS-NMR, the numerical integration method is performed to solve the modified Bloch equations under the longitudinal time-varying magnetic field and RF field co-excitation. Assuming that the amplitude of the longitudinal time-varying magnetic field B(t) is expressed as:

(2) $$B(t)=kB_{0}(e^{-m_{1}t}-e^{-m_{2}t})$$

where $B_{0}$ is the peak value of the time-varying magnetic induction intensity, the parameter k is an adjustment coefficient, $m_{1}$ and $m_{2}$ are the tuning parameters corresponding to the peak value appearing time. In the simulation process, the polarization direction of $\vec{B}(t)$ is along the +z axis, the duration time is 0.01 s and the peak value is 0.6 T (at the peak value, the corresponding Larmor frequency of the ^1^H nucleus is 25.55 MHz). The experimental sample is distilled water and its volume is $1.57\times 10^{-9}m^{3}$. The polarization direction of a modulated broadband RF excitation signal $\vec{B}(t)$ is along the +x axis. The magnetic flux density passing through the intersecting surface of the receiving coil varies with time and, thus, generates an induced electromotive force (EMF) in the solenoid coil. Since the longitudinal magnetic field, the RF excitation and the macroscopic magnetization $\vec{M}$ are all time-varying parameters, and there is no magnetic field along the y axis, the classic Bloch equations could be modified as Equation (3):

(3) $$\{\begin{array}{l} \frac{dM_{x}}{dt}=\gamma M_{z}(t)B_{z}(t)-\frac{M_{x}(t)}{T_{2}}\\ \frac{dM_{y}}{dt}=\gamma \left(M_{z}(t)B_{x}(t)-M_{x}(t)B_{z}(t)\right)-\frac{M_{y}(t)}{T_{2}}\\ \frac{dM_{z}}{dt}=-\gamma M_{y}(t)B_{x}(t)-\frac{M_{z}(t)-M_{z0}(t)}{T_{1}} \end{array}$$

where $T_{1}$ and $T_{2}$ are the longitudinal relaxation time and transverse relaxation time, respectively, and the parameter $M_{z0}(t)$ represents the magnetization component of the thermal equilibrium state before the RF excitation signal is applied. Additionally, the total magnetic induction intensity ${\vec{B}}_{total}(t)=\vec{B}(t)+{\vec{B}}_{1}(t)=B_{x}(t){\vec{a}}_{x}+B_{y}(t){\vec{a}}_{y}+B_{z}(t){\vec{a}}_{z}$ is satisfied in the above equations.

In order to acquire the instantaneous components $M_{x}(t)$, $M_{y}(t)$ and $M_{z}(t)$ of the macroscopic magnetization, the four-order Runge–Kutta (RK-4) method is adopted to solve the first-order linear, nonhomogeneous and variable coefficient differential equations. Before solving the equations, three conditions should be satisfied in advance. First of all, since the magnetic induction intensity of the longitudinal magnetic field is greater than that of the RF excitation field, the influence of the RF excitation on the longitudinal magnetic field could be ignored. Secondly, the spin relaxation time $T_{1}$ is independent from the magnetic induction intensity of the time-varying magnetic field $B_{z}(t)$ due to the narrow variation range of the time-varying magnetic field (~0.6T). Finally, the nuclear spin system should not be magnetized before the time-varying magnetic field is applied, which means that the macroscopic magnetization $M_{z0}(t)$ and $\vec{M}(0,0,0)$ are zeroes at the initial time t=0.

**Figure 1:**
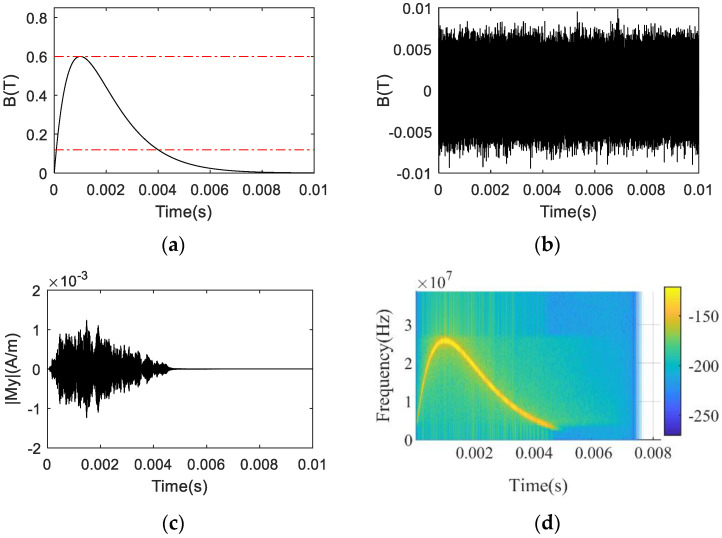
The numerical simulation results: (**a**) The waveform of the pulsed time−varying magnetic field; (**b**) The waveform of the RF excitation signal applied; (**c**) The waveform of the macroscopic magnetization component $M_{y}(t)$; (**d**) The STFT result of the macroscopic magnetization component $M_{y}(t)$.

In the process of the numerical simulation, the pulsed time-varying magnetic field and sinusoidal time-varying magnetic field are employed for performing the NSS-NMR experiment to verify the NSS-NMR effect. The former is an aperiodic magnetic field with dramatic changes and a short duration (about 0.01 s). The latter is a periodic time-varying magnetic field, whose amplitude and period (T = 0.02 s) are consistent with that of the mixed magnets in the actual experiments. In the numerical simulation, the excitation signal is a band-limited white noise signal, which is a Gaussian white noise signal that processed by an equal ripple band-pass filter. Suppose the Larmor precession frequency corresponding to the peak value $B_{0}$ of the pulsed time-varying magnetic field is $f_{0}$(=$\gamma B_{0}$), the parameters of the ripple band-pass filter utilized are as follows: the frequency range of the pass-band covers 0.2 $f_{0}$ to 1.0$f_{0}$ with the pass-band fluctuation as 0.01 dB, the transition band ranges from 0.1 $f_{0}$ to 0.2 $f_{0}$ and 1.0 $f_{0}$ to 1.1 $f_{0}$ respectively, and the stop-band attenuation is 80 dB. Figure 1 shows the numerical simulation results of the pulsed time-varying magnetic field; the black curve in Figure 1a is the waveform of the longitudinal pulsed time-varying magnetic field. Figure 1b is the waveform of the broadband RF excitation signal whose amplitude and frequency band are about 0.006 T and 20 MHz, respectively. Figure 1c describes the NSS-NMR time-domain response of the macroscopic magnetization component ${\vec{M}}_{y}(t)$, whose waveform is completely different from that of the conventional NMR response. It can be concluded from Figure 1c that the parameter ${\vec{M}}_{y}(t)$ is time continuing and lasts from 0.1 ms to 4.7 ms, which is consistent with the expected time duration of the NSS-NMR effect. Since the macroscopic magnetization increases as the magnetic induction intensity increases, the amplitude of ${\vec{M}}_{y}$ is correspondingly very weak at the beginning and end stages. Figure 1d displays the time–frequency diagram of the ${\vec{M}}_{y}$ obtained by short-time Fourier Transform (STFT), which is exactly in accordance with the time-varying magnetic field waveform shown in Figure 1a.

**Figure 2:**
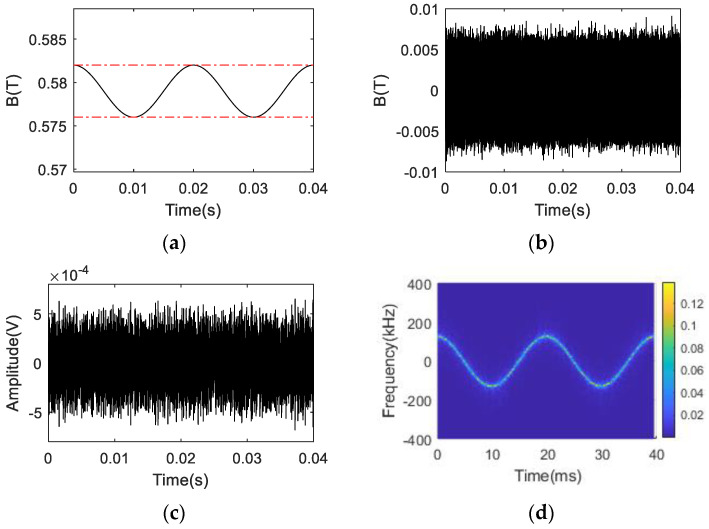
The numerical simulation results: (**a**) The waveform of the sinusoidal time−varying magnetic field; (**b**) The waveform of the RF excitation signal applied; (**c**) The waveform of the macroscopic magnetization component $M_{y}(t)$; (**d**) The STFT result of the macroscopic magnetization component $M_{y}(t)$.

The numerical simulation results of the NSS-NMR effect in the sinusoidal time-varying magnetic field are shown in Figure 2. Figure 2a is the waveform of the sinusoidal time-varying magnetic field with a period of 0.02 s, amplitude range of 0.576 T to 0.582 T and the data length selected for the simulation as0.04 s. The red dotted line represents the magnetic field range corresponding to the bandwidth of the RF excitation signal. It should be emphasized that the broadband RF excitation applied in the simulation is identical to that in the pulsed time-varying magnetic field numerical simulation. Figure 2c displays the NSS-NMR time-domain response of the macroscopic magnetization component ${\vec{M}}_{y}(t)$ of the sinusoidal time-varying magnetic field after digital down converters (DDC) due to its low frequency resolution. Figure 2c shows that the ${\vec{M}}_{y}(t)$ is time-varying and its duration is 0.04 s. Figure 2d describes the time–frequency diagram versus the offset frequency from the corresponding median value $B_{median}$ (=0.579 T) of the upper and lower ranges of the sinusoidal time-varying magnetic field. It can be seen from Figure 2d that a sinusoidal waveform is observed, which is consistent with the sinusoidal time-varying magnetic field waveform displayed in Figure 2a. The simulation of the above aperiodic and periodic time-varying magnetic field NMR experiment verifies the NSS-NMR effect and shows the feasibility of the magnetic field measurement by the NSS-NMR theory.

## 3. The NSS-NMR Experimental Scheme

### 3.1. The Time-Varying Magnetic Field and the Experimental System

**Figure 3:**
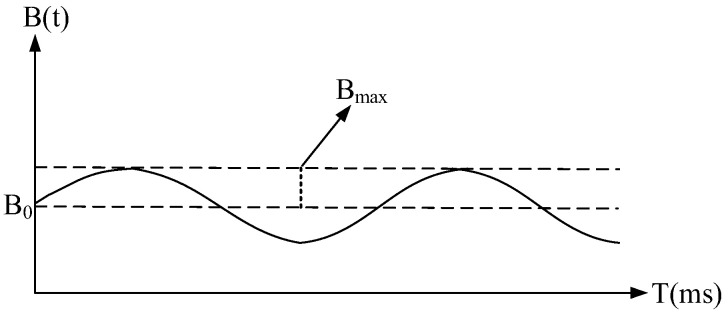
The waveform of the mixed magnet.

The time-varying magnetic field environment is generated by a mixed magnet, which includes a permanent magnet and an energized multi-turn Hermholtz coil connecting to a tunable AC voltage source. The AC voltage can be adjusted from 0 V to 250 V by a knob installed on top of the energized coil. Therefore, the intensity of the magnetic field of the mixed magnet could be controlled by the AC voltage source. The magnetic induction intensity of the mixed magnet could be expressed in Equation (4) as:

(4) $$B(t)=B_{0}+B_{\max}\cos(2\pi f_{0}t+\varphi_{0})$$

where $B_{0}$ corresponds to the magnetic induction intensity of the permanent magnet, and $B_{\max}$, $f_{0}$ and $\varphi_{0}$ represent the amplitude, frequency and initial phase of the magnetic induction intensity produced by the AC energized coil, respectively. The waveform of the time-varying magnetic field B(t) is shown in Figure 3, its DC component is about 0.579 T and the AC amplitude is about 0.003 T while the AC controlling voltage is set to 250 V. Corresponding to the magnetic induction intensity of the mixed magnet B(t), the Larmor precession frequency should cover from 24.526 MHz to 24.782 MHz for the ^1^H nuclei; therefore, the frequency bandwidth of the NMR probe should exceed at least 260 kHz.

**Figure 4:**
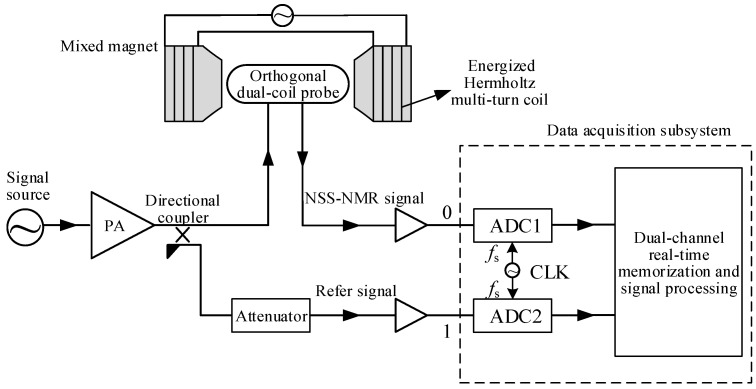
An NSS−NMR experimental system based on a mixed magnet, an orthogonal dual-coil probe and data acquisition subsystem.

An NSS-NMR experimental system mainly includes a mixed magnet, an RF broadband signal source, an orthogonal dual-coil probe, a directional coupler, an attenuator and a set of data acquisition subsystems, as displayed in Figure 4. The RF excitation signal, which is a band-limited white noise signal with a frequency bandwidth of 300 kHz that is generated by the signal source, is sent to the input of the directional coupler after amplified by the power amplifier (PA). The two-way output signals from the directional coupler feed into the transmitting coil of the orthogonal dual-coil probe and the reference receiving port “1” of the data acquisition subsystem, respectively. The NSS-NMR signal, the leakage part of the transmitting signal and the noise outputting from the induction coil of the probe feed into port “0” of the data acquisition subsystem. The port “1” signal can serve as a reference signal for the following subsequent signal processing. The sampling rate is 100 MHz and the total acquisition time is set to 50 ms to cover multiple periods of the longitudinal time-varying magnetic field.

### 3.2. The Probe Design and Signal Processing Scheme

#### 3.2.1. The Coil Structure Optimization

The signal-to-noise ratio (SNR) of an NMR signal is related to the quality of the NMR spectroscopy. In a conventional NMR experiment, the nuclear spin system is excited by a 90° pulse sequence [18,19], and the SNR of the free induction attenuation (FID) signal is illustrated in Equation (5).

(5) $$\begin{array}{ll} SNR=\frac{Peak\;Signal}{RMS\;Noise} & =\frac{k_{0}\omega_{0}{}^{2}(B_{1}/i)V_{s}N\gamma \hbar I(I+1)/3\sqrt{2}kT_{s}}{V_{n}}\\ & =\frac{k_{0}\omega_{0}{}^{2}(B_{1}/i)M_{0}V_{s}}{\sqrt{2}V_{n}} \end{array}$$

In the above equation, the SNR is relevant to the distribution homogeneity $k_{0}$ of the RF excitation field, the resonance frequency $\omega_{0}$, the excitation efficiency $B_{1}/i$, the macroscopic magnetization $M_{0}$, the sample volume $V_{s}$ and the noise RMS voltage $V_{n}$. Since the NMR signal is a macroscopic manifestation produced by the precession and nutation of all the spin nuclei with a non-zero magnetic moment in the sample space, the poor distribution homogeneity of the RF excitation field would eventually result in the gradual phase dispersion of the magnetization in the nutation plane [20,21]. The homogeneity parameter $k_{0}$ is related to many factors, such as the coil type, the length and diameter and the turn gap of the exciting/inducing coil, the gap between the coil and the wall of the magnet.

**Figure 5:**
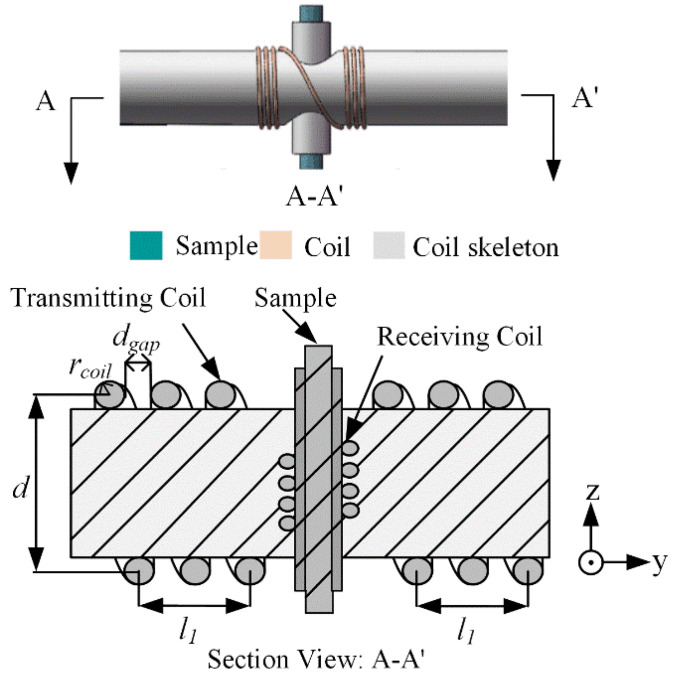
The section view of the orthogonal dual-coil probe.

A common coil, such as a solenoid, Helmholtz, saddle and birdcage coil, could be used to fabricate an NMR probe [22,23,24,25]. The solenoid coil possesses a higher excitation efficiency and better distribution homogeneity of the magnetic field [26]; therefore, it was selected to design the transmitting coil and the receiving coil of the orthogonal dual-coil installed in the NSS-NMR experimental system. The orthogonal placement of the transmitting coil and receiving coil could obviously increase the isolation between the two coils. As revealed in Figure 5, the ^1^H nuclear sample that is encapsulated inside a short tube is positioned in the internal area of the vertical polyformaldehyde (POM) stick. The solenoid coil wrapping around the vertical POM stick is defined as the receiving coil. The six-turn transmitting solenoid coil is divided into two parts and wrapped on both sides of a thicker horizontal POM stick. The diameter of the enameled wire is 0.5mm, and the net length of the transmitting solenoid is 4.5 mm.

Since the turn gap and the length-to-diameter ratio are two significant parameters that affect the RF excitation field homogeneity [27,28,29,30], the magnetic field homogeneity parameter $\delta_{B_{1}}$ is defined to quantify the magnetic field distribution homogeneity in the center region of the transmitting coil. Assuming that the total number of discrete grids on each coordinate axis is N, then the average value of the magnetic induction intensity in the center region of the RF excitation field can be expressed in Equation (6) as:

(6) $$B_{1mean}=\frac{1}{N}\left(\sum_{i}^{N_{x}}B_{1x,i}+\sum_{j}^{N_{y}}B_{1y,j}+\sum_{k}^{N_{z}}B_{1z,k}\right)$$

where $B_{1x,i}$, $B_{1y,j}$ and $B_{1z,k}$ are the magnetic induction intensity at the i-th grid along the x-axis, y-axis and z-axis, respectively, $N_{x}$, $N_{y}$ and $N_{z}$ are the numbers of the grids along each axis. Under the condition of i+j+k=N, the homogeneity of the RF excitation field $\delta_{B_{1}}$ is defined as Equation (7) as:

(7) $$\delta_{B_{1}}=\frac{1}{B_{1mean}}\sqrt{{\left(\frac{1}{N_{x}}\sum_{i}^{N_{x}}\left(B_{1x,i}-\frac{1}{N_{x}}\sum_{i}^{N_{x}}B_{1x,i}\right)\right)}^{2}+{\left(\frac{1}{N_{y}}\sum_{j}^{N_{y}}\left(B_{1y,j}-\frac{1}{N_{y}}\sum_{j}^{N_{y}}B_{1y,j}\right)\right)}^{2}+{\left(\frac{1}{N_{z}}\sum_{k}^{N_{z}}\left(B_{1z,k}-\frac{1}{N_{z}}\sum_{k}^{N_{z}}B_{1z,k}\right)\right)}^{2}}$$

**Figure 6:**
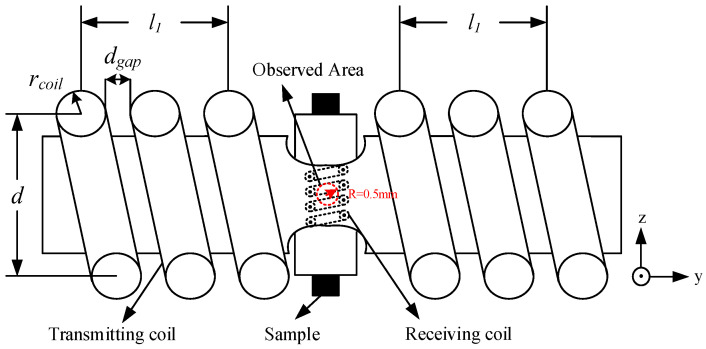
The front view of the observed area for the calculation of the RF excitation field homogeneity.

The above definition is the same as the standard deviation of the RF excitation field distribution. It means thatthe closer $\delta_{B_{1}}$ is to zero, the better homogeneity of the RF excitation field will be. In the case that the diameter, length and position of the transmitting coil is fixed, a different turn gap and length-to-diameter ratio should be observed for the influencing degree of the parameter $\delta_{B_{1}}$ while the observed area is set as a sphere with a diameter of 1 mm. The front view of the observed area for the calculation of the RF homogeneity is interpreted as the red dotted region in Figure 6.

The homogeneity $\delta_{B_{1}}$ of the RF excitation field corresponding to the turn gap is displayed in Table 1. It can be concluded that $\delta_{B_{1}}$ reaches a minimum value when the equation $d_{gap}=d_{coil}$ is established.

The homogeneity $\delta_{B_{1}}$ of the RF excitation field corresponding to the length-to-diameter ratio is shown in Table 2. The results show that $\delta_{B_{1}}$ reaches a minimum value while the length-to-diameter ratio l/d is 0.5 rather than the maximum ratio 0.8. The reason is that the two-part structure of the transmitting solenoid is more similar to a multi-turn Helmholtz coil.

#### 3.2.2. The Equivalent Circuit Parameters Extraction for the Orthogonal Dual-Coil Probe

**Figure 7:**
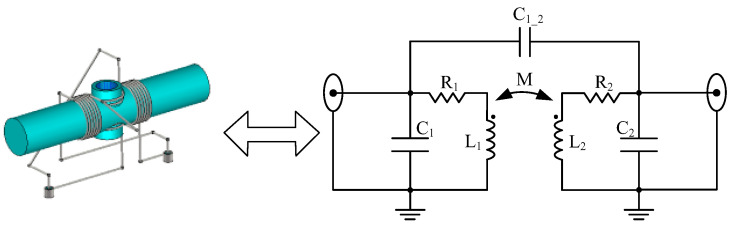
The equivalent lumped-parameter circuit of the orthogonal dual-coil probe.

The orthogonal dual-coil probe proposed in this paper consists of two mutually orthogonal solenoids; therefore, the equivalent lumped-parameter circuit could be depicted as Figure 7, where the mutual inductance M and mutual capacitance $C_{1\_2}$ between the two coils, the self-inductances $L_{1}$ and $L_{2}$, the turn capacitances $C_{1}$ and $C_{2}$ and the ohm resistance $R_{1}$ and $R_{2}$ are included in the equivalent circuit.

The above lumped-circuit parameters can be calculated by many analytical methods, such as the method of moments (MoM) [31], the boundary element method [32], the simulated charge method [33] and the finite element method (FEM) [34]. The MoM could be used to calculate the distribution inductance between the wire loops, but it depletes the amount of memory and time to solve the multiple loops inductance. The boundary element method could effectively reduce the number of variables, but it is not convenient to solve the electromagnetic field problems. The simulated charge method replaces the free charge that is continuously distributed on the electrode surface with the simulated charge, but the setting of the optimal position of the charge is normally determined by experience, resulting in a low calculation accuracy. The FEM can analyze the distribution inductance among any complex wire loops, and its calculation process is relatively simple. Therefore, the FEM combined with the electric field energy and the magnetic field energy is adopted to extract the equivalent lumped-circuit inductances and capacitances of the orthogonal dual-coil probe by applying the correct boundary condition to the simulation region.

Assume that the coil number of a multi-loop system is N, the loop current is $I_{i}$ (i=1,2,3…N), the total magnetic field energy of the multi-loop system $W_{m}$, as shown in Equation (8) as:

(8) $$W_{m}=\sum_{i=1}^{N}\frac{1}{2}\psi_{i}I_{i}==\int_{\tau }\frac{1}{2}\mu H^{2}d\tau$$

where H is the magnetic field intensity distributed in the whole region τ excited by the currents $I_{i}$ (i=1,2,3…N), $\psi_{i}$ is the total magnetic flux crossing over the i-th coil, as described in Equation (9) as:

(9) $$\psi_{i}=M_{i1}I_{1}+M_{i2}I_{2}+\cdots +M_{iN}I_{N}=\sum_{j=1}^{N}M_{ij}I_{j}$$

where $M_{ij}$ is the mutual inductance between the j-th (j=1,2,3…N) coil and the i-th coil, and $M_{ij}=M_{ji}$, $M_{ii}=L_{i}$ is established.

Therefore, the magnetic field energy $W_{m}$ can be expressed in Equation (10) as:

(10) $$\begin{array}{ll} W_{m}=\int_{\tau }\frac{1}{2}\mu H^{2}d\tau & =\sum_{i=1}^{N}\sum_{j=1}^{N}\left(\frac{1}{2}I_{i}M_{ij}I_{j}\right)\\ & =(\frac{1}{2}I_{1}{}^{2},\frac{1}{2}I_{2}{}^{2}\ldots \frac{1}{2}I_{N}{}^{2},I_{1}I_{2},I_{1}I_{3},\ldots ,I_{1}I_{N},I_{2}I_{3},I_{2}I_{4},\ldots ,I_{2}I_{N},\ldots ,I_{N-1}I_{N})\cdot \\ & {\left(L_{1},L_{2},\ldots ,L_{N},M_{12},M_{13},\ldots ,M_{1N},M_{23},M_{24},\ldots ,M_{iN},\ldots ,M_{N-1,}{}_{N}\right)}^{T} \end{array}$$

In the above equation, the total number of inductances to be solved is $Num=N+C_{N}^{2}=\frac{N(N+1)}{2}$. Therefore, $\frac{N(N+1)}{2}$ groups of loop currents $I^{\left(i\right)},i=1,2,\ldots ,\frac{N(N+1)}{2}$, should be set to excite the multi-loop system for calculating the magnetic field distribution $H^{\left(i\right)}$ and the corresponding energy $W_{M}^{\left(i\right)}$ in the whole space region. Where every exciting current group is $I^{\left(i\right)}=\left(I_{1}^{\left(i\right)},I_{2}^{\left(i\right)},I_{3}^{\left(i\right)}\ldots I_{N}^{\left(i\right)}\right),i=1,2,\ldots ,\frac{N(N+1)}{2}$, the magnetic field energy $W_{m}^{\left(1\right)},W_{m}^{\left(2\right)},\ldots ,W_{m}^{\left(i\right)},\ldots ,W_{m}^{\left(\frac{N(N+1)}{2}\right)}$, is as illustrated in the matrix Equation (11) below.

(11) $$\left(\begin{array}{c} W_{m}^{\left(1\right)}\\ \vdots \\ W_{m}^{\left(N\right)}\\ W_{m}^{\left(N+1\right)}\\ \vdots \\ W_{m}^{\left(\frac{N(N+1)}{2}\right)} \end{array}\right)=\left(\begin{array}{c} \int_{\tau }\frac{1}{2}\mu {\left(H^{\left(1\right)}\right)}^{2}d\tau \\ \vdots \\ \int_{\tau }\frac{1}{2}\mu {\left(H^{\left(N\right)}\right)}^{2}d\tau \\ \int_{\tau }\frac{1}{2}\mu {\left(H^{\left(N+1\right)}\right)}^{2}d\tau \\ \vdots \\ \int_{\tau }\frac{1}{2}\mu {\left(H^{\left(\frac{N(N+1)}{2}\right)}\right)}^{2}d\tau \end{array}\right)=\left(\begin{array}{cccccc} \frac{1}{2}{\left(I_{1}^{\left(1\right)}\right)}^{2} & \frac{1}{2}{\left(I_{2}^{\left(1\right)}\right)}^{2} & \cdots & I_{n}^{\left(1\right)}I_{n+1}^{\left(1\right)} & \cdots & I_{N-1}^{\left(1\right)}I_{N}^{\left(1\right)}\\ \vdots & \vdots & \ddots & \vdots & \ddots & \vdots \\ \frac{1}{2}{\left(I_{1}^{\left(N\right)}\right)}^{2} & \frac{1}{2}{\left(I_{2}^{\left(N\right)}\right)}^{2} & \cdots & I_{n}^{\left(N\right)}I_{n+1}^{\left(N\right)} & \cdots & I_{N-1}^{\left(N\right)}I_{N}^{\left(N\right)}\\ \frac{1}{2}{\left(I_{1}^{\left(N+1\right)}\right)}^{2} & \frac{1}{2}{\left(I_{2}^{\left(N+1\right)}\right)}^{2} & \cdots & I_{n}^{\left(N+1\right)}I_{n+1}^{\left(N+1\right)} & \cdots & I_{N-1}^{\left(N+1\right)}I_{N}^{\left(N+1\right)}\\ \vdots & \vdots & \ddots & \vdots & \ddots & \vdots \\ \frac{1}{2}{\left(I_{1}^{\left(\frac{N(N+1)}{2}\right)}\right)}^{2} & \frac{1}{2}{\left(I_{2}^{\left(\frac{N(N+1)}{2}\right)}\right)}^{2} & \cdots & I_{n}^{\left(\frac{N(N+1)}{2}\right)}I_{n+1}^{\left(\frac{N(N+1)}{2}\right)} & \cdots & I_{N-1}^{\left(\frac{N(N+1)}{2}\right)}I_{N}^{\left(\frac{N(N+1)}{2}\right)} \end{array}\right)\left(\begin{array}{c} L_{1}\\ \vdots \\ L_{N}\\ M_{1,2}\\ \vdots \\ M_{N-1,\;N} \end{array}\right)$$

The above matrix equation could be abbreviated as W=IM, so all the inductances could be solved from the equation $M=I^{-1}W$. Here, every magnetic energy $W_{m}^{\left(i\right)}$ is numerically integrated by using the magnetic field distribution $H^{\left(i\right)}$ calculated by the 3D FEM. It is very important to properly set up the value and direction of every loop current so as to guarantee the non-singularity of the matrix I.

The turn capacitances and the mutual capacitances of the dual-coil probe could also be solved by a similar method in the electric field environment. As a result, you only need to replace the current excitations with the multiple group charge excitations and calculate the electric field distribution by FEM and the energy density to calculate the electrostatic field energy under the different charge excitation groups. The extracted equivalent circuit parameters of the orthogonal dual-coil probe are displayed in Table 3. It can be concluded that the self-inductance, turn capacitance and ohm resistance values of the transmitting coil are all greater than those of the receiving coil, which accord with the facts that the turn number and diameter of the transmitting coil are all greater than those of the receiving coil. Moreover, the mutual electromagnetic coupling between the transmitting coil and the receiving coil is very weak due to the orthogonal placement of the coils reflected from the small mutual inductance and mutual capacitance, which indicates that the orthogonal dual-coil probe possesses a naturally high isolation between the input and output ports.

#### 3.2.3. Dual-Port Matching for the Orthogonal Dual-Coil Probe

Since the RF excitation signal is broadband and time continuous in the NSS-NMR experiment, it is necessary to cascade the two impendence matching circuits on the two ports of the orthogonal dual-coil structure. The basic requirement of the matching circuits is to broadly match the impendence of the T/R coils to the impendence of the external source or load on the premise of ensuring enough isolation between the two ports. Due to the limitation of the available space (L × W × H = 54 × 11 × 60 mm^3^) for placing the matching circuits inside of the mixed magnet, a remote matching strategy is utilized practically, i.e., two long coaxial lines that link the orthogonal dual coils and matching circuits are placed outside of the magnet.

There are two kinds of remote matching methods, i.e., remote resistance matching and remote reactance multiple Π-stage matching for the dual-coil probe. The former possesses a wide frequency band, high isolation and simple circuit but a weak current excitation efficiency of the RF magnetic field. The latter possesses a strong current excitation efficiency but a narrow frequency band, poor isolation, complicated circuit and painful debugging process. If the continuous-wave average output power of the RF exciting source is enough or affluent, the remote resistance matching circuits are recommended for achieving a wider frequency band and higher isolation. Otherwise, the remote reactance matching circuits should be designed to achieve a high excitation efficiency under the conditions of a lower average power output of the excitation source.

**Figure 8:**
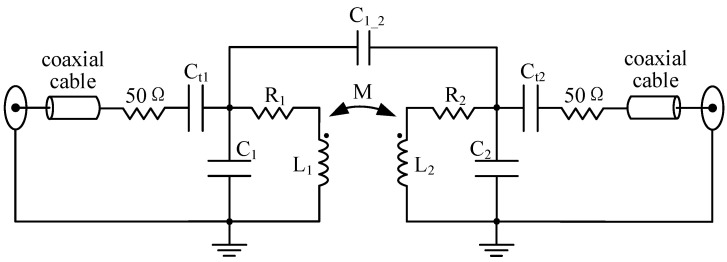
The remote resistance matching for the orthogonal dual-coil probe.

Following the simulation of the aforementioned equivalent lumped-parameter circuit of the orthogonal dual-coil probe, only a 50 Ohm resistor and a capacitor are required in the remote resistance matching circuit, as depicted in Figure 8. The capacitors $C_{t1}$ and $C_{t2}$ are for tuning the imaginary part of the input impedance of the T/R coils, respectively, and the two 50 Ohm resistors are employed to match the very small real part of the input impedance of the T/R coils to the source impedance or load impedance.

**Figure 9:**
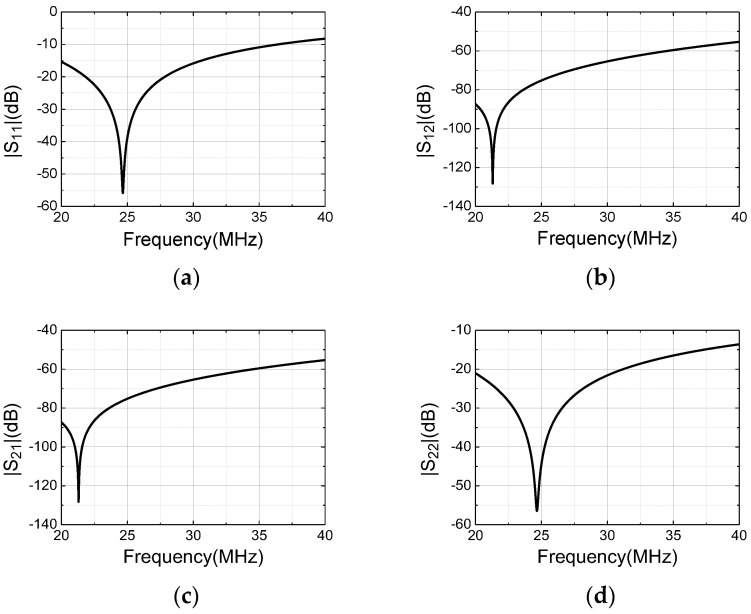
The S−parameters of the remote resistance matching for the orthogonal dual-coil probe (**a**) S_11_; (**b**) S_12_; (**c**) S_21_; (**d**) S_22_.

The practical performance of the remote resistance matching method is revealed in Figure 9. Figure 9a,d show that the 20 dB and 10 dB impedance matching bandwidths are 5 MHz and 12.77 MHz, respectively. Figure 9b,c show that the 60 dB isolation bandwidth between the T/R ports exceeds 15 MHz, at a center frequency of 24.66 MHz of the NSS-NMR experiment on the condition of the ^1^H nuclear sample and the average value 0.579 T of the mixed magnet.

**Figure 10:**
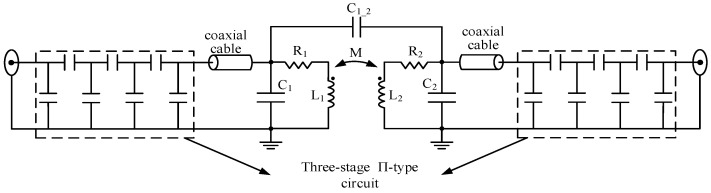
The remote reactance three-stage Π-type matching for the orthogonal dual-coil probe.

Figure 10 describes the remote reactance three-stage Π-type matching circuit, which is also designed and tuned practically for the actual probe prototype. Since the one-stage capacitance Π-type matching circuit only possesses a narrow bandwidth, the three-stage Π-type circuit is used in each remote port of the probe [35].

**Figure 11:**
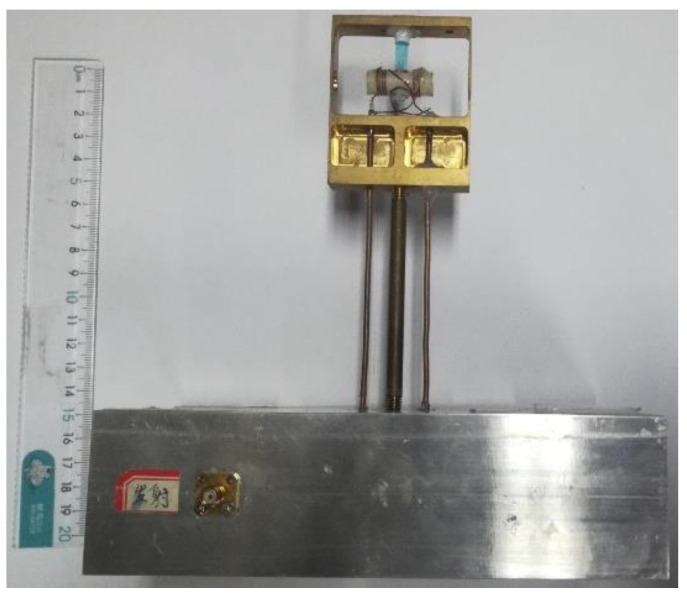
The prototype of the orthogonal dual-coil probe with remote reactance matching circuits.

The prototype of the proposed wideband orthogonal dual-coil probe with the remote reactance matching circuits is devised and fabricated, as shown in Figure 11. The probe consists of a coil skeleton, nuclear sample, transmitting coil, receiving coil and matching circuits. Meanwhile, a thickness of up to 5 mm copper baffle (not shown in the figure) is designed to reduce mutual coupling between the outside T/R matching circuits.

Due to the non-magnetism of the resistors and capacitors, the latter matching circuits could also be installed in neighboring positions of the dual-coil structure when the inner space of the magnet is roomy enough. This near matching circuit may achieve a better performance and wider bandwidth than the remote one.

**Figure 12:**
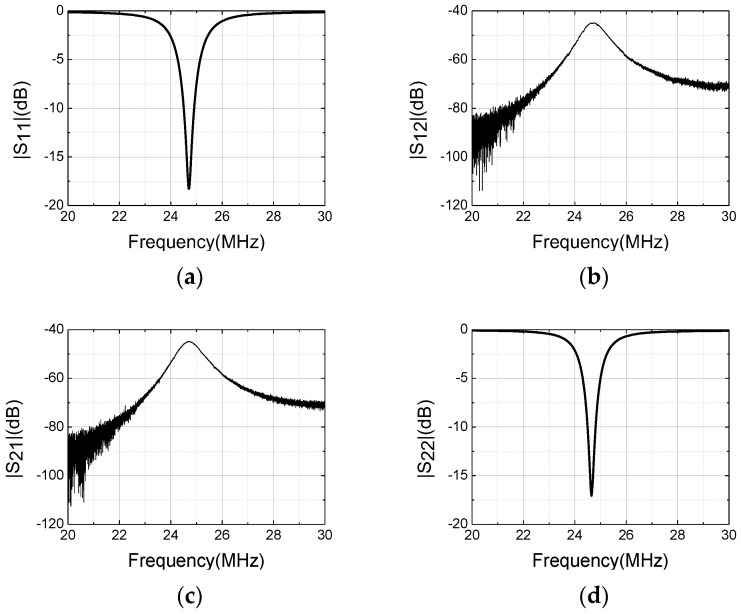
The measurement performances of the prototype probe: (**a**) S_11_; (**b**) S_12_; (**c**) S_21_; (**d**) S_22_.

The S-parameter measurement results of the prototype probe are presented in Figure 12. The transmitting coil is connected to port 1 and the receiving coil is connected to port 2 of the vector network analyzer (VNA). A 10-dB impedance matching bandwidth is about 300 kHz, which coversfrom 24.50 MHz to 24.80 MHz. The isolation between the T/R ports is greater than 45.09 dB, which indicates that the performance of the very weak leakage from the RF exciting port to the receiving port will help to easily extract the NSS-NMR signal from the output compound signal in the subsequent signal processing process.

#### 3.2.4. Signal Processing Scheme

**Figure 13:**
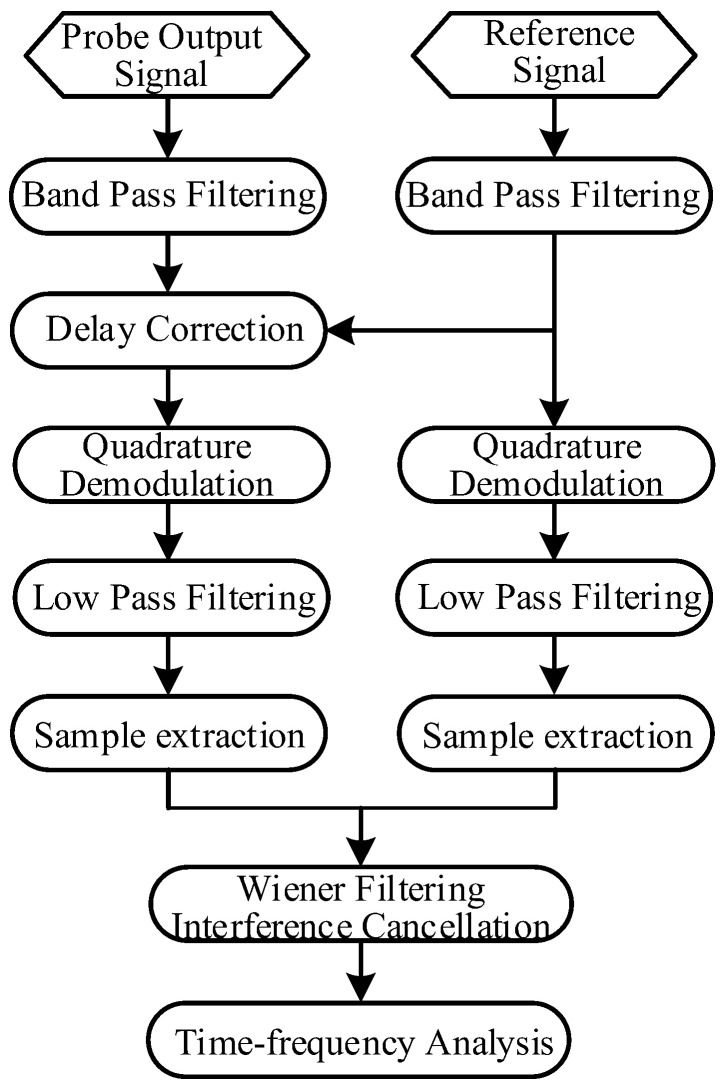
The signal processing scheme for achieving the NMR signal of the NSS-NMR effect.

Due to the finite isolation between the T/R coils, there is a small part of the RF excitation signal leaking from the input port to the output port. Although the leakage is very small, it is still much greater than the power of the NSS-NMR signal. Therefore, it is necessary to cancel the RF excitation leakage from the compound of the output signal to finally extract the NSS-NMR signal. As a result, a Wiener adaptive filtering interference cancellation algorithm is used in the data processing scheme. The reference signal comes from the coupling port of the directional coupler. The overall signal processing scheme includes band-pass filtering (BPF), delay correction, quadrature demodulation, low-pass filtering (LPF), sample extraction, adaptive interference cancellation (Wiener filtering) and time–frequency analysis, as depicted in Figure 13. The delay correction processing is for aligning the group-delay-time (GDT) of the probe output signal to that of the reference signal. The time-difference value corresponding to the peak of the cross-correlation function between the reference signal and output signal of the probe might be used to align the two signals. The quadrature demodulation, LPF and sample extraction are for outputting the digital baseband I/Q signals to conduct the subsequent adaptive filtering and time–frequency analysis.

## 4. Processing Results

**Figure 14:**
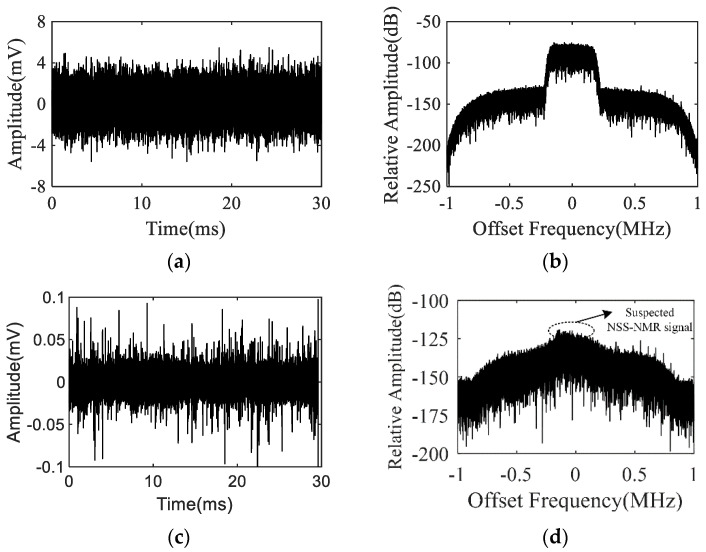
Results of: (**a**) The waveform of the original probe output signal. (**b**) The power spectrum of the original probe output signal. (**c**) The waveform of the probe output signal after interference cancellation by adaptive filtering. (**d**) The power spectrum of the probe output signal after interference cancellation.

Here, a segment of 30 ms time-length acquisition data with original sampling rate 100 MHz is utilized for verifying the NSS-NMR effect and the signal processing scheme. After quadrature demodulation, LPF and sample extraction, the sampling rate has been decreased to 2 MHz yet is covering the efficient frequency range of the NSS-NMR signal. Since the Wiener filter’s order is 800, the front 800 sampling points should be removed to avoid time–frequency analysis distortion. Figure 14 displays the waveform and corresponding power spectrum of the probe output signal before and after the interference cancellation. According to the time- and frequency-domain results, the interference cancellation degree of the Wiener filtering algorithm is about 46 dB. The residuary part in Figure 14d shows the suspected NSS-NMR signal.

**Figure 15:**
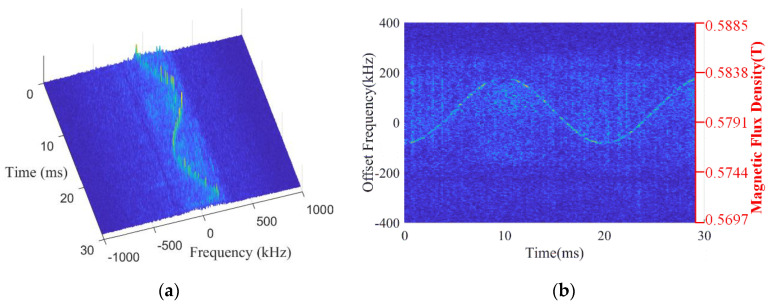
The time−frequency analysis results: (**a**) The 3D STFT diagram. (**b**) The inverted magnetic flux density of the mixed magnet vs. time variation.

After the interference cancellation, the processed data are divided into 228 sub-segments for carrying out STFT. Each sub-segment consists of 1024 sampling points with 75% overlapped points. The time–frequency diagram of the data of these segments versus the offset frequency from the NMR frequency corresponding to parameter $B_{0}$ is displayed in Figure 15a. A sinusoidal waveform could be clearly observed, which demonstrates the existence of the NSS-NMR effect, and the NSS-NMR signal has been successfully extracted. In addition, when the excitation RF power is low, the NSS-NMR signal is also weak in certain time segments. The inverted magnetic flux density of the mixed magnet by the Larmor precession relationship is shown in Figure 15b, which indicates that the range of time-varying magnetic field range is from 0.576 T to 0.582 T and the time-varying period is 20 ms. The experimental results are identical to the actual parameters of the mixed magnet. There is only a slight difference in the display clarity between the simulation and the actual experiment, which is mainly due to the spectral peak of the weak signals in certain segments submerged by the background noise.

## 5. Conclusions

A novel NSS-NMR effect is forecasted, and an NSS-NMR experimental scheme is proposed to verify the principle. In the NSS-NMR experiment, the nuclear spin system is excited by the continuous broadband RF signal; therefore, the excited NSS-NMR signal is composed of numerous signal components with different frequencies and amplitudes which vary with time. The NSS-NMR effect could be used to invert the magnetic induction intensity of the longitudinal time-varying magnetic field by the Larmor precession relationship. The NSS-NMR signal and the magnetic field inversion are successfully validated in a mixed sinusoidal time-varying magnet environment.

The next validation experiment would be carried out in a pulsed high magnet environment, where a more compact orthogonal dual-coil probe and more efficient matching circuits are needed to accommodate the new requirements. The NSS-NMR effect is even expected to provide a new observation method for studying the field-induced phase transition phenomenon and material structure with broad spectrum characteristics in a pulsed high magnetic field environment. In addition, the proposed NSS-NMR effect may be applied in material analysis (such as ^27^Al and ^29^Si nuclei in clay minerals) and liver disease detection.

## Figures and Tables

**Table 1:** Homogeneity of the RF excitation field corresponding to the turn gap.

*d*_gap_	0.5 *r*_coil_	*r*_coil_	1.5 *r*_coil_
$\delta_{B_{1}}$	0.0095	0.0056	0.0102

**Table 2:** Homogeneity of the RF excitation field corresponding to the length-to-diameter ratio.

l/d	0.2	0.4	0.5	0.6	0.8
$\delta_{B_{1}}$	0.011	0.0154	0.0103	0.0155	0.0318

**Table 3:** The extracted equivalent circuit parameters of the orthogonal dual-coil probe.

Parameters	Self- Inductance (Unit: μH)	Turn Capacitance (Unit: pF)	Ohm Resistance of Coil (Unit: mΩ)	Mutual Inductance (Unit: nH)	Mutual Capacitance (Unit: pF)
Transmitting Coil	0.2625	1.145	18.7	0.29	0.449
Receiving Coil	0.1373	0.491	8.6		

## Data Availability

Not applicable.
